# Genetic Regulation of ERK1/2–c-Fos Signalling Pathway in Newcastle Disease Virus–Induced Apoptosis of Human Pancreatic Cancer Stem Cells

**DOI:** 10.21315/mjms-08-2025-607

**Published:** 2026-02-28

**Authors:** Seyed Abbas Ghasemi, King-Hwa Ling, Saila Ismail, De-Ming Chau, Zamberi Sekawi

**Affiliations:** 1 Department of Medical Microbiology, Faculty of Medicine and Health Sciences, Universiti Putra Malaysia, Serdang, Selangor, Malaysia; 2 Department of Biomedical Sciences, Faculty of Medicine and Health Sciences, Universiti Putra Malaysia, Serdang, Selangor, Malaysia; 3 Department of Biotechnology and Bimolecular Sciences, Faculty of Biotechnology and Bimolecular Sciences, Universiti Putra Malaysia, Selangor, Malaysia

**Keywords:** pancreatic adenocarcinoma stem cells, RAS molecular signalling pathway, Newcastle Disease Virus, gene expression profiling, apoptosis

## Abstract

**Background:**

Pancreatic ductal adenocarcinoma (PDAC) is a highly aggressive cancer with a five-year survival of approximately 6% that is largely driven by cancer stem cells (CSCs), which promote resistance, metastasis, and recurrence. Mutations in the Ki-ras2 Kirsten rat sarcoma viral oncogene homolog (K-RAS) are common in human PDAC and activate the rat sarcoma (RAS) pathway, thus supporting tumour growth, maintenance, and metastasis. Newcastle Disease Virus (NDV) is a tumour-selective oncolytic virus with a strong safety profile that targets cancer cells and therapy-resistant CSCs, thereby highlighting its potential as an anticancer virotherapy. This study examines NDV AF2240’s ability to target CSCs through RAS pathway modulation and apoptosis induction.

**Methods:**

NDV AF2240 was titrated by hemagglutination (HA) and plaque assays using Vero cells. CSCs were isolated from Panc-1 cells and confirmed by sphere-formation, flow cytometry, and RT-qPCR for CD24, CD44, and EpCAM/ESA. Cells were infected at multiplicities of infection (MOIs) of 0.1, 0.5, 1, and 10 for 24–72 h. Cytopathic effects were assessed with an MTS assay, and apoptosis was assessed by the expression of Caspase-3, -8, and -9, Bax, and Bcl- 2. NDV reduced CSC viability in a time- and dose-dependent manner, with significant declines at 24–48 and 72 h (*P* < 0.05). Apoptosis markers Caspase-3, -8, and -9, and Bcl-2 were significantly upregulated (M = 3.4613, 5.9173, 2.4610, and 2.2083, respectively; *P* < 0.05). An MOI of 0.1 was used for pathway analysis in 1 × 10^6^ CSCs.

**Results:**

NDV AF2240 induced effective cytopathic effects in PDAC stem cells, downregulating the K-RAS effectors ERK1/2 and C-FOS, triggering apoptosis, and reducing the viability of PDAC CSCs in vitro.

**Conclusion:**

These findings highlight NDV’s potential as an oncolytic agent against PDAC stem cells via the modulation of ERK1/2 and C-FOS, thus supporting its therapeutic development.

## Introduction

Pancreatic cancer is a highly aggressive and deadly disease that ranks seventh among cancer-related deaths in developed countries (1). Pancreatic ductal adenocarcinoma (PDAC) stems from non-invasive epithelial growths in the pancreatic ducts and constitutes approximately 90% of all pancreatic cancers (2). Its high fatality rate is mainly because of late symptom onset and delayed diagnosis, with 458,918 new cases and 432,242 deaths reported worldwide in 2018 (3). In the United States, pancreatic cancer has had a five-year survival rate of below 5% for more than a decade (4). Moreover, its incidence has doubled over the past 20 years, increasing with age, and is higher in men (5.5/100,000) than in women (4.0/100,000) (3). The incidence of PDAC varies among countries, with the highest age-standardised incidence rates occurring in Europe and North America and the lowest rates in Africa and Central Asia (5). However, in Malaysia, pancreatic cancer is not among the top 10 most prevalent cancers. From 2012 to 2016, 2,049 cases were reported (1,167 men, 882 women), most commonly in Chinese males, followed by Malays and Indians, with the highest incidence in those aged ≥ 50 years (6).

Recent multimodal therapies offer limited survival benefits, mostly for patients with resectable tumours (7). Furthermore, relapse is often driven by a small (0.5% to 1.0%) population of hypoxic cancer stem cells (CSCs), known for their aggressive, metastatic nature (8). These CSCs resist conventional treatments by repairing DNA damage (9), expelling drugs, activating anti-apoptotic pathways (10), and surviving within a supportive tumour microenvironment of fibroblasts and immune cells (11).

RAS proteins are small GTPases that regulate cell growth, differentiation, and apoptosis and are among the most common genes that are mutated in human cancers. Of these, the K-RAS gene has the highest rate of missense mutations, which accounts for approximately 85% of RAS isoform mutations (12). Oncolytic viruses are emerging as effective cancer therapies, exhibiting promising results across various tumour types (13). Newcastle Disease Virus (NDV), or avian paramyxovirus type 1 (APMV-1), from the *Paramyxoviridae* family (14), targets cancer cells via sialic acid receptors while sparing healthy cells. Its HN glycoprotein binds to these receptors, which enables viral entry and release, while the F protein drives syncytium formation, thus aiding viral spread. NDV replicates in the cytoplasm without integrating into the host genome, leading to direct tumour cell lysis and the activation of interferons and tumour necrosis factor, all while maintaining a strong safety record in preclinical and clinical studies. Notably, its replication is enhanced by oncogenic RAS activation. Experimental evidence indicates that Rac1, a member of the Ras superfamily of GTPases, is essential for efficient NDV replication and its cytotoxic effects in specific human cancer cell models (15–17). This study explores how NDV affects the K-RAS signalling pathway in PDAC stem cells in vitro.

## Methods

### NDV AF2240 Titers

The neurotropic-viscerotropic velogenic NDV strain AF2240, kindly provided by Dr. Saila Ismail, Virology Lab, Faculty of Biotechnology and Biomolecular Sciences, Universiti Putra Malaysia, was propagated in specific-pathogen-free embryonated chicken eggs up to passage 7. The viral stock was tested and confirmed to be free of mycoplasma and other contamination before use in experiments. The virus was titrated by hemagglutination (HA) assay using fresh chicken red blood cells (18), and infectious particles were quantified by plaque assay on Vero cells and expressed as plaque-forming units (pfu) (19).

### Cell Culture and Stem Cell Isolation

Panc-1 cells (ATCC CRL-1469™) were maintained in Dulbecco’s Modified Eagle’s Medium (DMEM) with 10% fetal bovine serum (FBS), 100 U/mL of penicillin, and 100 μg/mL of streptomycin at 37°C in 5% CO_2_. When the cells had achieved 90% confluence, they were trypsinised, seeded at a density of 1000 cells/mL in DMEM/F-12 with growth supplements, and cultured in low-attachment flasks. Spheroid formation was evident from day 10 and propagated every 14 days. Morphological changes consistent with epithelial-to-mesenchymal transition were documented by inverted microscopy, and the resulting spheroid clusters were designated as Panc-1 CSCs (20).

### Stem Cell Identification

A CSC subpopulation in Panc-1 cells formed spheroids with self-renewal, proliferation, differentiation, and tumorigenic traits, which was confirmed by sphere assays, flow cytometry (CD24, CD44, and EpCAM), and RT-qPCR analysis for gene expression.

### Sphere-formation Assay

Panc-1 spheres were dissociated using 0.25% trypsin and 0.02% EDTA, neutralised with serum-free medium (SFM), and centrifuged at 300× g for 10 min. The pellet was suspended in 1 mL of SFM and diluted to 5 cells/mL, and 200 μL thereof was plated per well in a 96-well flat-bottom plate and incubated at 37°C with 5% CO_2_. The cell suspension was inspected under a microscope to confirm that the cells were well separated and did not form clumps. Individual stem cells were observed daily for 14 days, with sphere-formation visible within 10 to 14 days (21). Upon transfer to serum-containing medium (SCM), the spheres reverted to an epithelial-like morphology, which is consistent with mesenchymal-to-epithelial transition (MET) within two days (22).

### Flow Cytometry

Flow cytometry analysis was performed using anti-human CD24 (APC), CD44 (PE), and EpCAM/ESA (FITC) monoclonal antibodies (R&D Systems, USA). The antibodies used included anti-human CD24 allophycocyanin-conjugated mAb (clone ML5, Cat. No. GZFAB5247A), anti-human CD44 phycoerythrin-conjugated mAb (clone IM7.8.1R, Cat. No. GZ-FAB6127P-025), and anti-human EpCAM/TRoP1 fluorescein-conjugated mAb (clone 158206, Cat. No. GZ-FAB9601F). Panc-1 and Panc-1 stem cell suspension were aliquoted in tubes at a density of 5 × 10^5^ cells and stained for 30 min using a combination of anti-human CD24 APC (mouse IgG2A), anti-human CD44 PE (rat IgG2B), and anti-human EpCAM/ESA FITC (mouse IgG2B) on ice in the dark. The controls included single-stained samples, an unstained sample, and an isotype control to ensure accurate gating. Five microliters of each conjugated antibody was added to the respective tube, whereas a combined tube received a mixture of 5 μL of each antibody. The unstained tube was left without antibodies, whereas 5 μL of an isotype control antibody was added to the negative control tube. After staining, the cells were washed twice with phosphate-buffered saline containing 0.2% bovine serum albumin, suspended in 400 μL of staining buffer, and analysed using a BD FACS Canto II flow cytometer. Two-colour flow cytometry was performed with sequential gating of three markers, and the populations were quantified using FACS DIVA software (v6.1.2). As CD44-PE and EpCAM-FITC were both excited by the 488-nm blue laser, spectral overlap was corrected by fluorescence compensation, with an emission maximum of 575 nm for PE and 520 nm for FITC. CD24-APC required no compensation as it was excited by the red laser. For gating, the cells were first selected by forward and side scattering to exclude debris, followed by singlet discrimination. The PANC-1 cells were identified by CD44 (PE) positivity and subsequently analysed for EpCAM and CD24 expression, with the same strategy applied to the stem cell population. The complete gating workflow is presented in the result section (20).

### RNA Extraction and RT-qPCR Analysis of CD24, CD44, and EpCAM Genes

Total RNA was extracted from 1 × 10^6^ Panc-1 and Panc-1 stem cells using the innuPREP RNA Mini Kit and treated with an RNase inhibitor. The RNA quality was confirmed using a NanoDrop, with the A260/280 and A260/230 ratios close to 2.0. For cDNA synthesis, 500 ng of RNA was reverse transcribed using the Tetro cDNA Synthesis Kit with anchored-random hexamer primers in a 20 μL reaction with a thermocycle protocol of 25°C for 10 min, 45°C for 30 min, 85°C for 5 min, and hold at 4°C. Samples were stored at −20°C. RT-qPCR was performed using the SensiFAST SYBR Green Kit on a LightCycler^®^ 480 System in 20 μL reactions. The cycling conditions were 95°C for 2 min, followed by 40 cycles of 95°C for 5 s, 60°C for 10 s, and 72°C for 20 s, ending with a 30 s hold at 40°C. In addition, GAPDH and *β*-actin served as the internal controls. Each group included three biological replicates (*n* = 3), with each sample tested in duplicate. The relative gene expression levels of CD24, CD44, and EpCAM were calculated using the 2^−ΔΔCt^ method. The stability of GAPDH and *β*-actin was confirmed by their consistent Ct values across all samples and their well-established reliability as housekeeping genes, thus justifying their use for 2^−ΔΔCt^ normalisation. The primers presented in Table 1 were derived from published sources, and the corresponding citations have been included.

### MTS Cytotoxicity Assay of Stem Cells

The cytolytic effect of NDV AF2240 on Panc-1 stem cells was determined using the CellTiter 96^®^ AQueous One Solution Cell Proliferation Assay (Promega, Madison, WI, USA) according to the manufacturer’s protocol. Cells were seeded in 96-well plates at a cell density of 3 × 10^4^/well with SFM and incubated at 37°C with 5% CO_2_ for 24 h. Wells were then infected with 50 μL of NDV at multiplicities of infection (MOIs) of 0.1, 0.5, 1, or 10, while the controls received fresh medium. After 1 h, the media was replaced with 100 μL of fresh SFM. Cell viability was measured at 24, 48, and 72 h post-infection (hpi) by adding 20 μL of MTS reagent, incubating for 3 h to 4 h, and then measuring the absorbance at 490 nm using a TECAN Infinite^®^ F50 microplate reader (Magellan V7.2 SPI). Each MOI was tested in triplicate for each time point (27). The percentage cell viability was calculated by normalising the absorbance of treated cells to the corresponding uninfected control at the same time point, which was defined as 100% viability.

### RT-qPCR Analysis of Apoptosis-related Genes

RT-qPCR was employed to measure the mRNA levels of apoptosis-related genes, such as Caspase-3, -8, and -9, Bax, and Bcl-2, in NDV-treated and untreated Panc-1 stem cells. Total RNA was extracted from 1 × 10^6^ cells using the innuPREP RNA Mini Kit and treated with an RNase inhibitor. The RNA purity was confirmed using a NanoDrop 1000. cDNA was synthesised in 20 μL reactions using the SensiFAST SYBR Green kit. Amplification was performed on a Light Cycler 480 system using 10 μL of SYBR mix, 0.8 μL of each appropriate primer, 1 μL of cDNA, and RNase-free water up to 20 μL. The thermal cycling conditions were 95°C for 2 min, followed by 40 cycles of 95°C for 5 s, 60°C for 10 s, and 72°C for 20 s, and final cooling at 40°C for 30 s. GAPDH and *β*-actin were used as the internal controls. Each group included three biological replicates (*n* = 3), with two technical replicates performed per sample. Reactions were run in triplicate to ensure reproducibility. The relative expression was calculated using the 2^−ΔΔCt^ (Livak) method. The stability of GAPDH and *β*-actin was confirmed by their consistent Ct values across all samples as well as their well-established reliability as housekeeping genes, thus justifying their use for 2^−ΔΔCt^ normalisation. All primer sequences listed in Table 2 were obtained from previously published studies, and the corresponding references have been included.

### RT-qPCR Gene Expression Profiling Analysis of the K-RAS Molecular Pathway in Panc-1 Stem Cells

Panc-1 stem cells were seeded at a cell density of 1 × 10^6^ cells/well in 6-well plates with SFM and incubated at 37°C with 5% CO_2_ for 24 h. The cells were then infected with NDV at 0.1 MOI, whereas the controls received fresh SFM. After 1 h of gentle rocking, the medium was replaced, and the cells were incubated for a further 24 h. Total RNA was extracted using the innuPREP RNA Mini Kit and treated with an RNase inhibitor. RNA purity was confirmed using a NanoDrop, and 500 ng of RNA was used to synthesise cDNA in a 20 μL reaction with random hexamers and the Tetro cDNA Synthesis Kit. The reverse transcription conditions were 25°C for 10 min, 45°C for 30 min, and 85°C for 5 min. RT-qPCR was performed using SensiFAST

SYBR Green on a LightCycler 480 system. Each 20 μL reaction contained SYBR mix, 0.8 μL of each appropriate primer, 1 μL of cDNA, and RNase-free water up to 20 μL. The cycling conditions were 95°C for 2 min, followed by 40 cycles of 95°C for 5 s, 60°C for 10 s, 72°C for 20 s, and 40°C for 30 s. Each group included three biological replicates (*n* = 3), with two technical replicates performed per sample. GAPDH and *β*-actin were used as the internal controls, and the relative gene expression was calculated using the 2^−ΔΔCt^ method. The stability of GAPDH and *β*-actin was confirmed by their consistent Ct values across all samples, with no significant variation between treatment groups, thereby supporting their use for 2^−ΔΔCt^ normalisation. All primers listed in Table 3 were derived from published sources, and the appropriate citations have been included.

### Statistical Analysis

Statistical computations were performed using IBM SPSS software version 25 on the Windows operating system platform. One-way and two-way analysis of variance (ANOVA) were employed to evaluate differences across groups, and Duncan’s multiple-range test (*P* < 0.05) was utilised to compare the group means. In addition, gene expression differences between groups were assessed using a one-sample *t*-test. All values were presented as the mean standard error, with a significance threshold set at *P* ≤ 0.05.

## Results

### Virus HA Assay

**Figure 1:**
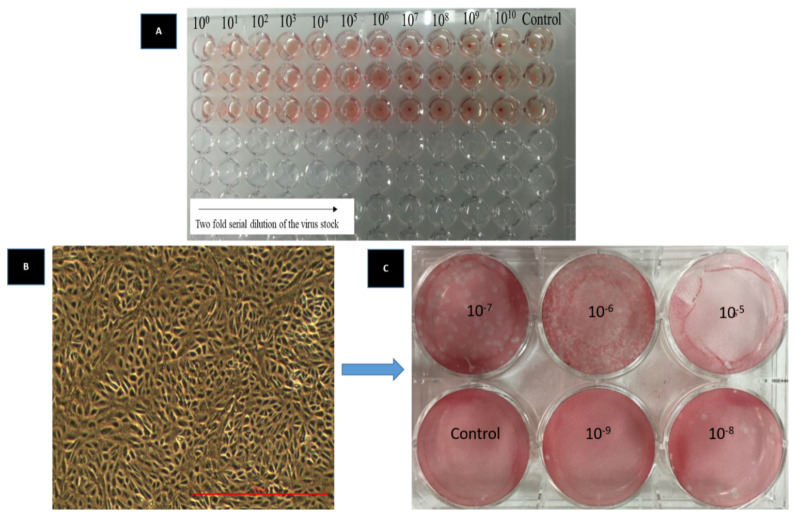
Evaluation of Newcastle Disease Virus (NDV) infectivity and titer using hemagglutination and plaque formation assays a) Viral hemagglutination activity was indicated by the formation of a uniform lattice, whereas the absence of a red button in the wells confirmed the presence of NDV at quantifiable titers b) Vero cell line (40× magnification). The red scale bar represents 0.05 μm c) Plaque assay results demonstrate the infectivity of NDV in Vero cells. Following infection with serial dilutions of NDV and incubation for 1 h, the removal of the inoculum and application of an overlay medium facilitated controlled viral propagation. After six days of incubation at 37°C, plaque formation was observed and quantified, thereby confirming successful viral replication and spread

The HA assay measured NDV’s ability to agglutinate red blood cells using twofold serial dilutions. Positive wells revealed diffuse films (agglutination), whereas the negative wells formed red dots at the bottom. The highest dilution with visible agglutination was 2^5^, which corresponds to a titer of 32 HA units per 50 μL. This correlates to a final viral concentration of 6400 HAU/μL (6.4 × 10^3^ HAU/mL) (Figure 1a).

### Virus Plaque Assay

A plaque formation assay was performed after the HA test to quantify the NDV particles using Vero cells. By day 6, plaques appeared in all infected wells except the control. The cells were fixed, stained with neutral red, and observed, where viable cells retained the stain, but the dead cells remained clear. At the 10^8^ dilution (well 4), plaque counts were within the countable range (20–100), with 25, 20, and 22 plaques recorded across the triplicates (mean = 22). The viral titer was determined to be 2.2 × 109 pfu/mL and used to calculate the MOI for subsequent Panc-1 stem cell infections.

### Stem Cell Isolation and Identification

**Figure 2:**
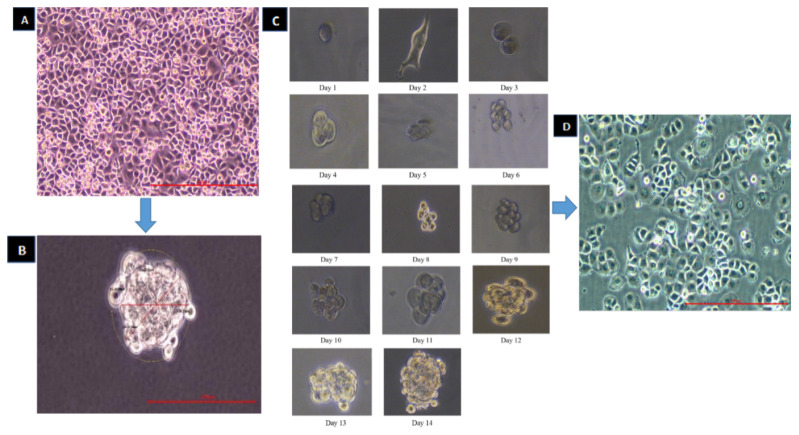
Workflow illustrating Panc-1 cell culture, cancer stem cell (CSC) isolation, and identification using the sphere-formation assay, followed by the characterisation of mesenchymal-to-epithelial transition (MET) a) Panc-1 cell monolayer (40× magnification). Panc-1 cells cultured in serum-containing medium (SCM) achieved confluence after 2 to 3 days, exhibiting characteristic epithelial morphology. The red scale bar represents 0.05 μm b) Panc-1 sphere (100× magnification). Images were acquired using an inverted microscope and captured with a digital camera (Olympus, Japan). The quantitative analysis was performed using ToupView software (Toup Tek, China) c) Sphere-formation assay. Within 10 to 14 days, spheroid formation was observed, thus demonstrating their ability to aggregate and proliferate in a serum-free, semi-adherent environment d) Illustration of MET of Panc-1 spheres within two days when grown in SCM. The transition was visualised at 4× magnification. The red scale bar represents 0.05 μm

Panc-1 cells were cultured in SCM, and the sphere-forming stem-like subpopulations were isolated and characterised (Figure 2a, b).

#### Sphere-formation Assay

Single cells from Panc-1 spheres formed clonogenic spheroids under serum-free conditions, thus demonstrating the capacity for self-renewal and proliferation. At low density (5 cells/mL), distinct spheroids developed within 10 to 14 days, thereby confirming their stem-like and tumorigenic potential. When recultured in SCM, these cells underwent MET, acquiring a polygonal, cobblestone-like morphology with enhanced cell–cell adhesion and compact colony formation within two days. The sphere-formation efficiency and morphological changes are shown in Figure 2c and d.

#### Flow Cytometry

**Figure 3:**
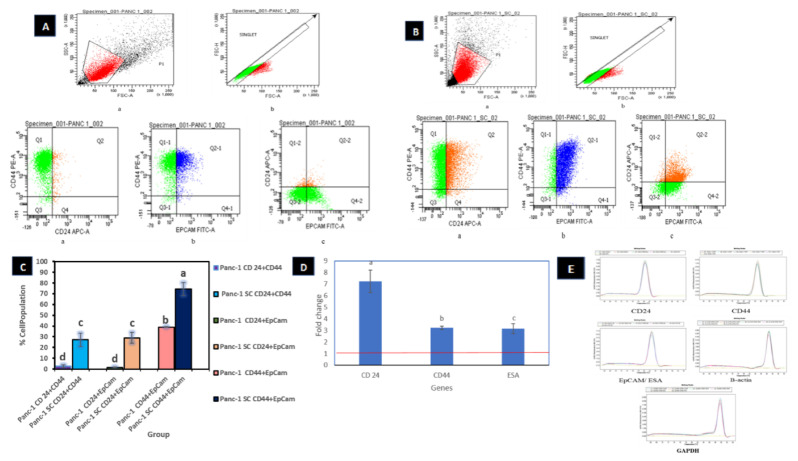
Integrated flow cytometry analysis and transcriptomic profiling of CD24, CD44, and EpCAM markers in Panc-1 and Panc-1 stem cell populations a) Representative flow cytometry dot plots demonstrating the gating strategy for Panc-1 cell populations. Scatter plot and single cells population followed by represented dot plots of Panc-1 double marker populations for CD44-PE vs. CD24-APC, CD44-PE vs. EpCAM-FITC, and CD24-APC vs. EpCAM-FITC b) Representative flow cytometry dot plots showing the gating strategy for Panc-1 stem cell population. Scatter plot and single cells population followed by representative dot plots of Panc-1 stem cell double marker population for CD44-PE vs. CD24-APC, CD44-PE vs. EpCAM-FITC, and CD24-APC vs. EpCAM-FITC c) Bar plot showing flow cytometry analysis of double-positive marker expression (CD24^+^/CD44^+^, CD24^+^/EpCAM^+^, and CD44^+^/EpCAM^+^) as percentages in the Panc-1 and Panc-1 stem cell groups. Data are presented as the mean (SD). Different letters above the bars (a–d) indicate significant differences between groups (*P* < 0.05) d) Bar plot showing fold changes in the expression of CD24, CD44, and EpCAM genes in Panc-1 stem cells compared to those of the control group. Each group included three biological replicates (*n* = 3), with each biological replicate analysed in duplicate (i.e., two technical replicates). Expression values were normalised to the internal control housekeeping genes GAPDH and *β*-actin and are presented as fold changes relative to Panc-1 cells (the hypothetical red reference line on the y-axis). Data are presented as the mean (SD). Different letters above the bars (a–c) indicate significant differences between groups (*P* < 0.05). Among the analysed genes, CD24 revealed the highest expression (M = 7.23) and EpCAM the lowest (M = 3.15) (*P* < 0.05) e) Melting curve analysis demonstrated a single distinct peak at 86°C, thereby confirming the specificity of the amplified product

Flow cytometry analysis demonstrated a significant enrichment of CD24^+^/CD44^+^ and CD24^+^/EpCAM^+^ double-positive populations in Panc-1 stem cells compared with the parental Panc-1 cells (*P* < 0.05). Specifically, CD24^+^/CD44^+^ cells increased from 2.67% in parental cells to 27.07% in stem cells, while the CD24^+^/EpCAM^+^ cells increased from 1.53% to 28.87% (*P* < 0.05). In addition, the CD44^+^/EpCAM^+^ population was markedly elevated in stem cells (74.40%) compared with that of the Panc-1 cells (38.90%), representing the most abundant marker combination in both cell types. Among all marker combinations, CD24^+^/CD44^+^ exhibited the lowest expression in stem cells, whereas CD24^+^/EpCAM^+^ was the least expressed in the parental Panc-1 cells (Figure 3a–c).

#### RT-qPCR Analysis of Markers in Panc-1 and Panc-1 Stem Cells

RT-qPCR data were statistically analysed following the confirmation of normal distribution and homogeneity of variance. A one-way ANOVA revealed significant differences in the expression levels of CD24, CD44, and EpCAM genes among the cell populations (F = 14.881, *P* = 0.005). Duncan’s multiple-range test further confirmed the significant upregulation of these stemness-associated markers in Panc-1 stem cells (*P* < 0.05), with CD24 exhibiting the highest expression (M = 7.23) and EpCAM the lowest (M = 3.15). In addition, a one-sample *t*-test indicated a significant difference in gene expression between Panc-1 stem cells and their parental Panc-1 counterparts (*P* < 0.05), thus supporting the elevated expression of stem cell markers and confirming the distinct molecular profile of Panc-1 stem cells (Figure 3d, e).

### Sensitivity of Stem Cells to NDV Infection

**Figure 4:**
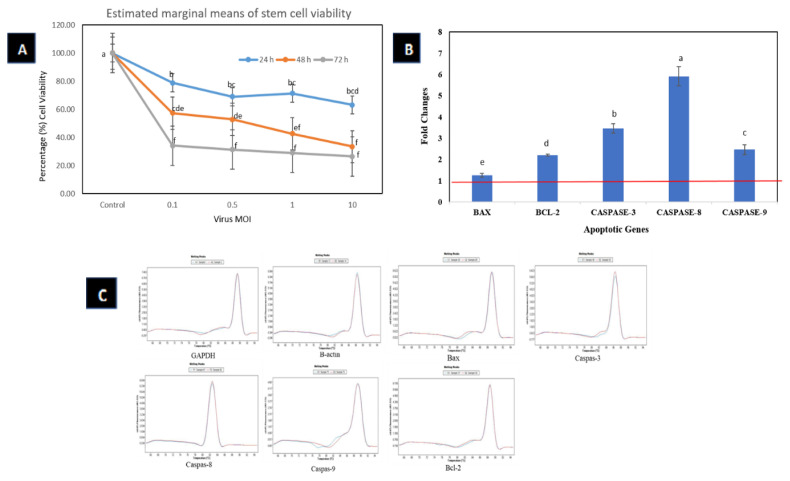
Graph, chart and bar plot showing the MTS cell viability assay and relative mRNA expression levels of apoptotic genes in Newcastle Disease Virus (NDV)-treated vs. untreated Panc-1 stem cells a) The graph illustrates the stem cell viability at 24, 48, and 72 hours post-infection (hpi) following NDV treatment at multiplicities of infection (MOIs) of 0.1, 0.5, 1, and 10 for the different viral concentrations. All experiments were performed in three biological replicates (*n* = 3). An MOI of 10 induced the highest cytotoxicity across all time points. Infection with an MOI of 0.1 led to significant cell death (78.89% viability) within 24 hpi, thus highlighting the potent oncolytic effects of NDV even at low concentrations b) Bar plots showing fold changes in the expression levels of intrinsic and extrinsic apoptotic genes. Data are presented as the mean (SD). Different letters above the bars (a–e) indicate significant differences between groups (*P* < 0.05). Among the analysed genes, Caspase-8 exhibited the highest expression level (M = 5.92) (*P* < 0.001), with a modest increase observed in the expression of Bax (M = 1.26). A one-sample *t*-test confirmed that these expression changes were significantly different from those in the untreated control cells (*P* < 0.05) c) The melt curve analysis, with a single peak for Caspase-3 at 82°C and at 88°C for the other genes, confirmed primer specificity

Results from the MTS assay confirmed that NDV AF2240 reduced the viability of human PDAC stem cells in a dose- and time-dependent manner compared to that of the untreated controls. A two-way ANOVA revealed a highly significant difference in cell viability among the various MOIs (0.1, 0.5, 1, and 10) at 24, 48, and 72 hpi (*P* < 0.001). According to Duncan’s post hoc test, MOI 10 consistently demonstrated the strongest cytotoxic effect, with the lowest cell viability recorded at 24 hpi (M = 63.15 ± 0.15), 48 hpi (M = 33.36 ± 10.19), and 72 hpi (M = 26.4 ± 6.05). Notably, even the lowest viral dose (0.1 MOI) resulted in a progressive and significant decline in stem cell viability over time, lowering from 78.89 ± 11.23% at 24 hpi to 57.22 ± 7.97% at 48 hpi and reaching 34.11 ± 1.67% at 72 hpi (*P* < 0.05). At 24 hpi, the cell viability values for MOIs of 0.1, 0.5, 1, and 10 were 78.89%, 68.93%, 71.35%, and 63.15%, respectively, thus indicating an early cytotoxic response even at low viral loads. An MOI of 0.1 was selected for pathway analysis, as it induced measurable cytotoxicity while retaining a sufficient number of viable cells for downstream RNA extraction and molecular analyses (Figure 4a).

### Confirmation of Stem Cell Death via Gene Expression Analysis of Caspase-3, -8, and -9, Bax, and Bcl-2

RT-qPCR data were analysed statistically following the confirmation of normal distribution and homogeneity of variance. A one-way ANOVA revealed a significant overall difference in gene expression among the treatment groups (F = 49.012, *P* < 0.001). Post hoc analysis using Duncan’s test revealed that NDV AF2240 treatment significantly upregulated Caspase-3 (M = 3.46), Caspase-8 (M = 5.92), Caspase-9 (M = 2.46), and Bcl-2 (M = 2.21) (*P* < 0.001), with a modest increase observed in the expression of Bax (M = 1.26). Furthermore, a one-sample *t*-test confirmed that these expression changes were significantly different from those in the untreated control cells (*P* < 0.05) (Figure 4b, c).

### Expression Analysis of the K-RAS Signalling Pathway Genes

**Figure 5:**
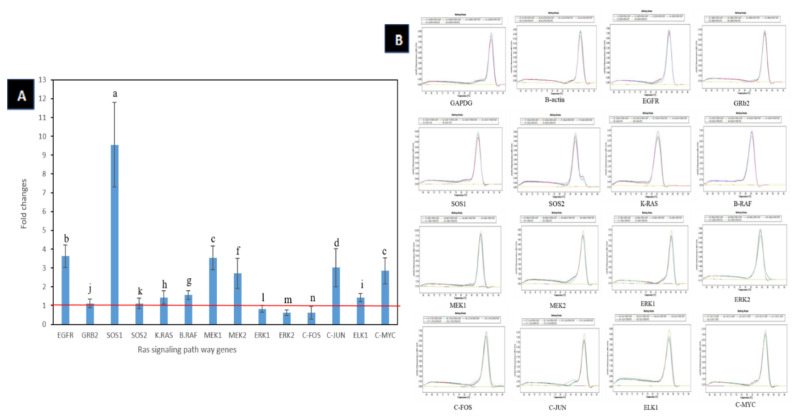
Relative mRNA expression levels of K-RAS and its upstream and downstream pathways in treated and untreated Panc-1 (SC), as determined by RT-qPCR Each group included three biological replicates (*n* = 3), with each biological replicate analysed in duplicate (i.e., two technical replicates). The expression values were normalised to the internal control housekeeping genes GAPDH and *β*-actin and are presented as fold changes relative to the untreated Panc-1 stem cells (the hypothetical red reference line on the y-axis) a) Bar plots showing fold changes in the expression of K-RAS and its effector genes. Data are presented as the mean (SD). Different letters above the bars (a–n) indicate significant differences between groups (*P* < 0.05). Among the analysed genes, SOS1 exhibited the highest relative expression, whereas ERK1, ERK2, and C-FOS showed the lowest. These findings suggest that infection with the Newcastle Disease Virus (NDV) AF2240 strain may suppress critical downstream effectors of the RAS signalling pathway, which may be associated with the induction of apoptosis b) Melt curve analysis of the K-RAS upstream and downstream genes reveals a single peak at 86°C, thus confirming primer specificity

After confirming the normal distribution and homogeneity of variance, RT-qPCR data of K-RAS pathway gene expression in NDV AF2240–treated Panc-1 stem cells were statistically compared with those of the untreated controls. ANOVA analysis demonstrated a significant difference in gene expression between the various genes in the treatment group (F = 9.899, *P* < 0.001). Duncan’s post hoc test revealed significant differences in the expression of MEK1, EGFR, and SOS1 (M = 3.5470, 3.6267, and 9.5523, respectively), in which SOS1 exhibited the highest expression (M = 9.5523) among the analysed genes, whereas ERK1, ERK2, and C-FOS showed the lowest expression levels (M = 0.8230, 0.6307, and 0.6173, respectively). Moreover, GRB2, SOS2, ELK1, K-RAS, and B-RAF displayed expression levels close to baseline (M = 1.1150, 1.1150, 1.4290, 1.4337, and 1.5573, respectively). In contrast, MEK2, C-MYC, and C-JUN were relatively upregulated (M = 2.7190, 2.8500, and 3.0187), but their expression levels declined over time (*P* < 0.05). A one-sample *t*-test comparing the means of the treated and untreated groups confirmed that ERK1/2 and C-FOS were downregulated in the treatment group. The suppression of these genes was associated with the induction of apoptosis and subsequent cell death, which suggests potential clinical relevance. These results indicate that NDV AF2240 interferes with K-RAS signalling and promotes cell death by modulating the downstream effectors, as shown in Figure 5a and b.

## Discussion

NDV AF2240 was successfully propagated and quantified using HA and plaque assays, thereby confirming its biological activity and replication competence. The HA titer of 32 HAU/50 μL and plaque formation on Vero cells (2.2 × 10^9^ pfu/mL) are consistent with previously reported NDV propagation efficiency (18, 41, 42). These results validate the viral stock’s suitability for downstream functional studies.

Pancreatic CSCs were successfully enriched from Panc-1 cells using sphere-formation, flow cytometry, and RT-qPCR. The formation of spheroids within 10 to 14 days, together with the ability of single dissociated cells to regenerate as spheres in SFM and revert to an epithelial phenotype upon re-exposure to SCM within two days, demonstrates their strong self-renewal capacity and phenotypic plasticity through MET (21, 22).

Furthermore, flow cytometry revealed the significantly higher expression of CD24^+^/CD44^+^, CD24^+^/EpCAM^+^, and CD44^+^/EpCAM^+^ subpopulations in CSCs compared with those of the parental Panc-1 cells (20, 43, 44), with CD44^+^/EpCAM^+^ being the dominant phenotype. These findings were further supported by RT-qPCR analysis, which revealed the significant upregulation of CD24, CD44, and EpCAM in CSCs (45, 46), thus confirming successful CSC enrichment and a distinct stem-like molecular profile.

NDV AF2240 induced strong dose- and time-dependent cytotoxicity in Panc-1 CSCs. While higher MOIs produced maximal cell death, an MOI of 0.1 induced progressive viability reduction over time and was selected for pathway analysis to preserve sufficient viable cells for molecular studies (27, 47). At 72 hpi, cytotoxic saturation was observed across the MOIs, thus indicating a strong overall oncolytic effect.

Gene expression profiling demonstrated that NDV modulated the K-RAS/MAPK/ERK signalling cascade. Although EGFR remained moderately active, further downstream signalling was attenuated through reduced GRB2 and SOS2 activity, while elevated SOS1 was partially sustained via upstream signalling (48). K-RAS and B-RAF exhibited only marginal activation, and MEK1/2 was moderately expressed, which indicates an overall weakened signalling cascade (49). Key downstream transcriptional regulators, including ELK1, C-JUN, and C-MYC, were reduced, thereby suggesting the suppression of prosurvival and proliferative signalling. The marked ERK1/2 reduction and downregulation of C-FOS suggest impaired prosurvival signalling in PDAC stem cells, as apoptosis was promoted. The expression levels of ERK1/2 and C-FOS were reduced after NDV infection, indicating biologically or clinically relevant downregulation. Although these changes were not significant, they likely reflect a shift in gene expression toward apoptosis, which is consistent with NDV-induced cell death (49–51). The ERK/C-FOS pathway is well-established as a driver of pancreatic tumour progression and therapy resistance. ERK1/2 activation regulates anti-apoptotic targets, such as Bcl-2, and contributes to gemcitabine resistance in pancreatic cancer (52), whereas ERK inhibition disrupts the tumour–stroma interactions and metastasis (50). Furthermore, You et al. (53) demonstrated that ERK/C-FOS signalling promotes the progression of pancreatic intraepithelial neoplasia, thus reinforcing the therapeutic relevance of ERK pathway suppression.

NDV infection also activated the intrinsic and extrinsic apoptotic pathways, as shown by the significant upregulation of Caspase-3, -8, and -9, Bax, and Bcl-2. Caspase-8 elevation indicates death receptor-mediated extrinsic apoptosis, whereas the increased Caspase-9 expression reflects mitochondrial pathway activation that is regulated by BCL-2 family proteins. Moreover, Caspase-3 activation confirms execution-phase apoptosis (54). Interestingly, Bcl-2 was upregulated alongside pro-apoptotic markers. Although Bcl-2 is classically considered anti-apoptotic, it has been reported to exert proapoptotic effects under specific cellular stress conditions and may serve as a dual regulator depending on the protein interactions and conformation (53, 55, 56). The concurrent increase in Bcl-2, Bax, and caspases, together with the cytotoxicity data, suggests the activation of intrinsic and extrinsic apoptotic pathways. Overall, these findings indicate a complex, context-dependent regulation of apoptosis in response to NDV treatment and warrant further mechanistic studies to clarify the precise role of Bcl-2. This study used a single PDAC cell line and an in vitro model, and the expression levels of ERK1/2 and C-FOS were measured at the mRNA level. Despite providing valuable mechanistic insights, future work, including protein-level analyses, in vivo models, and nonmalignant control cells, would better define NDV’s effects and tumour selectivity.

## Conclusion

In summary, NDV AF2240 effectively targets Panc-1 CSCs in vitro, induces apoptosis, and modulates the key components of the K-RAS/ERK signalling pathway. Although these findings do not demonstrate in vivo efficacy, they do support NDV AF2240 as a promising candidate for further preclinical evaluation against pancreatic CSCs. Although SOS1 expression remains elevated, its effect on K-RAS signalling appears partial and context-dependent, thus indicating that it is insufficient to fully sustain the activation of this pathway. This study highlights the potential of NDV AF2240 as a therapeutic agent against pancreatic CSCs and supports the future exploration of combination strategies, such as SOS1 inhibition, to potentially enhance the efficacy of anticancer therapies.

## Figures and Tables

**Table 1:** Sequences of the primers for CD24, CD44, EpCAM/ESA, and housekeeping genes used for RT-qPCR

Genes	Primers	Nucleotide sequence	Size (nt)	Reference
CD24	F	5′-GAAAACTGAATCTCCATTCCACA-3′	24	(23)
	R	5′-TGAAGAACATGTGAGAGGTTTGAC-3′	24	

CD44	F	5′-AGAAGGTGTGGGCAGAAGAA-3′	20	(24)
	R	5′-AAATGCACCATTTCCTGAGA-3′	20	

EpCAM/ESA	F	5′-GTGCTGGTGTGTGAACACTG-3′	20	(25)
	R	5′-GAAGTGCAGTCCGCAAACTT-3′	20	

GAPDH	F	5′-TTGGTATCGTGGAAGGACTCA-3′	21	(26)
	R	5′-TGTCATCATATTTGGCAGGTT-3′	21	

*β*-actin	F	5′-ACCAACTGGGACGACATGGAG-3′	21	(24)
	R	5′-GTGAGGATCTTCATGAGGTAGTC-3′	23	

**Table 2:** Sequences of the primers for Caspase-3, -8, and -9, Bax, Bcl-2, and housekeeping genes used for RT-qPCR

Genes	Primers	Nucleotide sequence	Size (nt)	Reference
Caspase-3	F	5′-TGCCTGTAACTTGAGAGTAGATGG-3′	24	(28)
	R	5′-CTTCACTTTCTTACTTGGCGATGG-3′	24	

Caspase-8	F	5′-GAT CAA GCC CCA CGA TGA C-3′	20	(29)
	R	5′-CCT GTC CAT CAG TGC CAT AG-3′	20	

Caspase-9	F	5′-CAT TTCATG GTG GAG GTG AAG-3′	21	(29)
	R	5′-GGG AAC TGC AGG TGG CTG-3′	18	

Bax	F	5′-ATC CAG GAT CGA GCA GGG CG-3′	20	(28)
	R	5′-GGT TCT GAT CAG TTC CGG CA-3′	20	

Bcl-2	F	5′-CATCAGGAAGGCTAGAGTTACC -3′	22	(28)
	R	5′-CAG ACA TTC GGA GAC CAC AC-3′	20	

GAPDH	F	5′-TTGGTATCGTGGAAGGACTCA-3′	21	(26)
	R	5′-TGTCATCATATTTGGCAGGTT-3′	21	
*β*-actin	F	5′-ACCAACTGGGACGACATGGAG-3′	21	(24)
	R	5′-GTGAGGATCTTCATGAGGTAGTC-3′	23	

**Table 3:** Sequences of the primers for K-RAS pathway-related and housekeeping genes used for RT-qPCR

Genes	Primers	Nucleotide sequence	Size (nt)	Reference
EGFR	F	5′-GGATGCCGACGAGTACCTC-3′	19	(30)
	R	5′-GCTTTGCAGCCCATTTCTAT-3′	20	

SOS1	F	5′-TGAGAGGCAACAGAAAGAGC-3′	20	(31)
	R	5′-GAGAAGGGAAATGAAATGGG-3′	20	

SOS2	F	5′-CCGCAGCCTTACGAGTTCTTC-3′	21	(32)
	R	5′-GGATGCACTTGTTCCTGAACC-3′	21	

GRB2	F	5′-ATTCCTGCGGGACATAGAACA-3′	21	(33)
	R	5′-GGTGACATAATTGCGGGGAAAC-3′	22	

K-RAS	F	5′-GAGGCCTGCTGAAAATGACTG-3′	21	(34)
	R	5′-ATTACTACTTGCTTCCTGTAGG-3′	22	

B-RAF	F	5′-TGATTTTGGTCTAGCTACAGT-3′	21	(35)
	R	5′-TGAATAAGGTAACTGTCCAG-3′	20	

MEK1	F	5′-CAGAAGAAGCTGGAGGAGCTAG-3′	22	(36)
	R	5′-CCATCGCTGTAGAACGCACCAT-3′	22	

MEK2	F	5′-CCAAGGTCGGCGAACTCAAA-3′	20	(37)
	R	5′-TCTCAAGGTGGATCAGCTTCC-3′	21	

ERK1	F	5′-TCAGACTCCAAAGCCCTTGACCT-3′	23	(36)
	R	5′-AAGCGTGCTGTCTCCTGGAAGAT-3′	23	

ERK2	F	5′-ATGCTGACTCCAAAGCTCTGGACT-3′	24	(36)
	R	5′-TCTGAGCCCTTGTCCTGACAAATT-3′	24	

ELK1	F	5′-CCTTCTATCAGCGTGGAT-3′	18	(38)
	R	5′-GTGGTGGTAGTAGTAGTC-3′	18	

C-FOS	F	5′-CCGGGGATAGCCTCTCTTAC-3′	20	(39)
	R	5′-GTGGGAATGAAGTTGGCACT-3′	20	

C-JUN	F	5′-TCAGACAGTGCCCGAGATG-3′	19	(40)
	R	5′-CTGCTGCGTTAGCATGAGTT-3′	20	

C-MYC	F	5′-CAAACCTCCTCACAGCCCACT-3′	21	(34)
	R	5′-TGACACTGTCCAACTTGACCC-3′	21	

GAPDH	F	5′-TTGGTATCGTGGAAGGACTCA-3′	21	(26)
	R	5′-TGTCATCATATTTGGCAGGTT-3′	21	

*β*-actin	F	5′-ACCAACTGGGACGACATGGAG-3′	21	(24)
	R	5′-GTGAGGATCTTCATGAGGTAGTC-3′	23	
